# IL-1β Promotes Vasculogenic Mimicry of Breast Cancer Cells Through p38/MAPK and PI3K/Akt Signaling Pathways

**DOI:** 10.3389/fonc.2021.618839

**Published:** 2021-05-14

**Authors:** Muhammad Azhar Nisar, Qin Zheng, Muhammad Zubair Saleem, Bulbul Ahmmed, Muhammad Noman Ramzan, Syed Riaz Ud Din, Naeem Tahir, Shuai Liu, Qiu Yan

**Affiliations:** ^1^Liaoning Provincial Core Lab of Glycobiology and Glycoengineering, College of Basic Medical Science, Dalian Medical University, Dalian, China; ^2^Department of Pathology and Pathophysiology, College of Basic Medical Science, Dalian Medical University, Dalian, China; ^3^Department of Microbiology, College of Basic Medical Science, Dalian Medical University, Dalian, China

**Keywords:** breast cancer, vasculogenic mimicry, interleukin-1 beta, VE-cadherin, VEGFR-1

## Abstract

Vasculogenic mimicry (VM), a micro vessel-like structure formed by the cancer cells, plays a pivotal role in cancer malignancy and progression. Interleukin-1 beta (IL-1β) is an active pro-inflammatory cytokine and elevated in many tumor types, including breast cancer. However, the effect of IL-1β on the VM of breast cancer has not been clearly elucidated. In this study, breast cancer cells (MCF-7 and MDA-MB-231) were used to study the effect of IL-1β on the changes that can promote VM. The evidence for VM stimulated by IL-1β was acquired by analyzing the expression of VM-associated biomarkers (VE-cadherin, VEGFR-1, MMP-9, MMP-2, c-Fos, and c-Jun) via western blot, immunofluorescent staining, and Immunohistochemistry (IHC). Additionally, morphological evidence was collected via Matrigel-based cord formation assay under normoxic/hypoxic conditions and microvessel examination through Hematoxylin and Eosin staining (H&E). Furthermore, the STRING and Gene Ontology database was also used to analyze the VM-associated interacting molecules stimulated by IL-β. The results showed that the expression of VM biomarkers was increased in both MCF-7 and MDA-MB-231 cells after IL-1β treatment. The increase in VM response was observed in IL-1β treated cells under both normoxia and hypoxia. IL-1β also increased the activation of transcription factor AP-1 complex (c-Fos/c-Jun). The bioinformatics data indicated that p38/MAPK and PI3K/Akt signaling pathways were involved in the IL-1β stimulation. It was further confirmed by the downregulated expression of VM biomarkers and reduced formation of the intersections upon the addition of the signaling pathway inhibitors. The study suggests that IL-1β stimulates the VM and its associated events in breast cancer cells via p38/MAPK and PI3K/Akt signaling pathways. Aiming the VM-associated molecular targets promoted by IL-1β may offer a novel anti-angiogenic therapeutic strategy to control the aggressiveness of breast cancer cells.

## Introduction

Breast cancer is the second most common cancer type, with an incident rate of 12.5% among women 5 years ago (1). Despite all the advances in cancer research and therapies, proliferation and metastasis remain a major problem contributing to the reoccurrence, drug resistance, and poor prognosis. It is generally considered that the rich blood supply is a determinant factor in cancer malignancy. Studies have been done related to this field, highlighting the underlying molecular mechanisms associated with tumor growth and perfusions 15 years ago (2). Due to unsatisfactory results from angiogenic inhibitors used in clinical trials suggests further exploration. Therefore, discovering the molecular mechanisms of angiogenesis and finding a way to efficiently arrest the angiogenesis is of great significance in inhibiting cancer malignancy and improving the overall survival of patients.

The new findings provide evidence that there exists an independent microvascularization process in cancer growth and perfusion (2). Except the classical angiogenesis originated from the endothelial cells of the blood vessel, the new type of angiogenesis, directly from the transformed cancer cells, was found in the microenvironment of the cancer tissues, which is called vasculogenic mimicry (VM) (3). During VM development, cancer cells acquire malignant characteristics, such as invasiveness, poor differentiation, and mesenchymal phenotypes, etc., resulting in tubule, cord, network-like or microvessel-like structures. Meantime, the expressions of many vascular-associated markers are elevated, such as vascular endothelial cadherin (VE-cadherin), vascular endothelial growth factor receptor-1 (VEGFR-1), MMP-9, and MMP2. Although VM is independent of the classical angiogenesis, the research data have shown that the overall angiogenesis of cancer can achieve a higher peak because of the involvement of VM, which aggravates the aggressive behaviors of cancer cells (4) and lead to poor clinical outcomes (5, 6). VM has been found in many cancer types, such as prostate cancer, ovarian cancer, and lung cancer. The underlying mechanism of VM in breast cancer remains unelucidated (7, 8).

Interleukin-1 beta (IL-1β) is a pro-inflammatory cytokine that plays critical regulatory roles in many physiological and pathological processes (9–11). IL-1β is upregulated in many types of cancers, including breast cancer, suggesting its potential role in facilitating tumorigenesis (11–13). The property of IL-1β to initiate inflammatory stress is considered due to the p38/MAPK signaling pathway (14). The relation of IL-1β mediated p38/MAPK activation in VM is not well-characterized. The stress-related responses can lead to the activation and the expression of several protein biomarkers associated with micro-transformations (15). It has been established that IL-1β is capable of regulating angiogenesis (16, 17). Proteome profiling reveals that IL-1β plays similar roles as VEGF does in human umbilical vein endothelial cells by activating the MAPKs signaling pathway (18). IL-1β is reported to play a role in activating c-Jun N-terminal kinase in cardiac myocytes (19). Furthermore, it can also promote angiogenic responses in cardiac myocytes by upregulating the expression of VEGF and VEGFR-2 (20). Moreover, the report suggests that IL-1β also plays a role in regulating the stabilization of hypoxia inducible factor alpha (HIF-α) associated protein, targeting the induction of angiogenesis (21). A study indicates that IL-1β has a positive capability to promote angiogenesis at the early angiogenic response (13). Studies on the role of IL-1β in promoting VM in breast cancer cells have not been well-characterized, which could help us explore and develop effective anti-cancer therapeutic strategies.

Angiogenic regulatory factors are mostly focused on established tumors, which pose a limitation toward the evaluations of molecular interactions at initial phases. Therefore, it is necessary to address these early cellular responses against factors that might influence the invasive nature of cancer cells. In the current study, we gathered evidence regarding the effect of IL-1β on VM in MCF-7 and MDA-MB-231 breast cancer cells, by analyzing cord formations on Matrigel and evaluating the expression of VM biomarkers. Results showed that IL-1β promoted VM by upregulating the expression of VM biomarkers (VEGFR-1, VE-cadherin, MMP-9, and MMP-2) via activating p38/MAPK and PI3K/Akt signaling pathways. Targeting the VM elicited by IL-1β may offer a novel anti-angiogenic therapeutic strategy to control the malignancy of breast cancer cells.

## Materials and Methods

### Reagents and Antibodies

Recombinant human IL-1β (HZ-1164) was purchased from Proteintech Group (Wuhan, China). Cell counting kit-8 (CCK-8) was purchased from Beyotime Biotechnology (Shanghai, China). Matrigel was obtained from Corning Inc. USA. p38 inhibitor (SB203580) and PI3K inhibitor (GDC-0941) were acquired from Selleck Chemicals (Shanghai, China). Antibodies:VEGFR-1(#13687-1-AP), p38 (#14064-1-AP), MMP-9 (#10375), MMP-2 (#10373-2-AP), GAPDH (#10494-1-AP), and HIF-1α (#20960-1-AP) were obtained from Proteintech (Wuhan, China), c-Fos (#4384), p-c-Fos (S32, #5348), c-Jun(#9165), p-c-Jun (S73, #9164), were purchased from Cell Signaling Technology, p-p38 (#AB4822) and IL-1R1 (#Ab106278) from Abcam USA, p-PI3K (#BS4605), PI3K (#AP0230), Akt (#BS1810), p-Akt (T308, #AA331) was obtained from Bioworld Technology (Nanjing, China), VE-cadherin (#E-AB-12772) from Elabscience. HRP-conjugated goat anti-rabbit secondary antibody was purchased from ZSGB-BIO (Beijing, China).

### Cell Culture

Human breast cancer cell lines (MCF-7 and MDA-MB-231) acquired from the American Type Culture Collection (ATCC) (Beijing Zhongyuan limited, China). Cells were routinely cultured in Dulbecco's Modified Eagle's medium (DMEM) Basic (Gibco, USA) with 10–15% fetal bovine serum (FBS) (GIBCO, USA), supplemented with 100 units/ml of penicillin and 100 μg/ml of streptomycin (TransGene Biotech, China). Cultures were maintained at 37°C with circulating humidified 5% filtered CO_2_. Subculturing was regularly performed after 2–3 days, depending on the cell confluency.

### Tumor Model

All the procedures were performed following the ethical guidelines provided by Dalian Medical University's Specific Pathogen Free (SPF) Animal Care Center. Female BALB/c Nude mice (*n* = 3 per group) were acquired at the age of 8–10 weeks, weighing 18–20 g. Twelve hours day/light and free access food and water were provided. Breast cancer cells (MDA-MB-231) suspended in 0.2 ml of PBS were subcutaneously injected at the right flank. The tumor was developed, and mice were grouped randomly. One microgram/gram/day of IL-1β was injected in the high dose group, and 0.25 μg/g/day IL-1β was injected in the low dose group. On the 10th day of treatment, the tumor masses were removed and processed for further experiments.

### Immunohistochemistry and H&E Staining

The IHC and H&E staining were performed in paraffin-embedded slices (4 μm each) of the removed tumor tissues from control and treated mice. The slides were deparaffinized in xylene and then rehydrated using standard procedures followed by passing through graded alcohol. Slides for H&E and IHC were then separated. For IHC staining, hydrogen peroxide was applied to block the endogenous peroxidase activity, and then blocking was performed with goat serum at 37°C for 30 min. Primary antibody was added and incubated at 4°C overnight. The slides were then incubated with secondary antibody for 1 h in a humidified chamber. The slides were washed and incubated with a streptavidin peroxidase complex. Diaminobenzidine (DAB) was used as a signal visualizer. Brief hematoxylin staining was performed at the end, and the slide gave a yellowish-brown color for positive cells. For H&E stain was applied following standard procedures. The slides were washed via several alcohol changes to remove traces of water and rinsed with xylene. Images were taken using the microscope BX51, Olympus, Tokyo, Japan.

### Cell Proliferation and Cytotoxicity by CCK-8 Assay

Inhibitory concentration (IC_50_) value was assessed by performing proliferating and cytotoxicity assay using CCK-8 kit following the manufacturer's instructions. Briefly, 1 ×10^4^ cells in a 96-well plate were treated with different indicated concentrations of cobalt chloride (CoCl_2_) and incubated at 37°C overnight. Then, 10 μl of CCK-8 solution was added into each well. Cells were then incubated for 3–4 h, and then the optical density at 450 nm was measured using a microplate reader (BioTek Instruments, Winooski, USA).

### Western Blot

Cells were first washed with ice-cold PBS three times and then incubated on ice with RIPA lysis buffer. Total protein from cell lysates was collected and quantified by Coomassie blue assay using bovine serum albumin (BSA) as a standard. The quantified protein was loaded and separated in 8–12% SDS-PAGE gel and then transferred electrophoretically onto the nitrocellulose membrane. Blocking was performed by incubating the nitrocellulose membrane in 5% fat-free milk dissolved in TBST for 2–3 h. After washing in TBST, the membranes were incubated in the appropriately diluted primary antibody in TBST (1:500–1:1,000) at 4°C overnight. Membranes were then washed with TBST three times and incubated with HRP-conjugated secondary antibody. Enhanced electrochemical luminescence (ECL) system (Bio-Rad Laboratories, Hercules, CA, USA) was used to detect protein bands. The relative quantification of a target protein was analyzed by comparing band intensity with reference to GAPDH as an internal control using ImageJ software.

### Immunofluorescent Staining

MCF-7 and MDA-MB-231 cells were cultured on glass cover slides. Cells were treated with 15 ng/ml of IL-1β to detect VE-cadherin and VEGFR-1, p-c-Fos and p-c-Jun. Cells were washed with ice-cold PBS and fixed with 4% paraformaldehyde (PFA) for 10 min, followed by permeabilization in PBS containing 1% Triton X-100. Cells were incubated in a blocking solution containing 1% BSA and 22.52 mg/ml glycine for 30 min. After wash, cells were then incubated with the primary antibody in a humidified chamber at 4°C overnight. The slides were then washed and incubated with TRITC-conjugated goat anti-rabbit IgG and FITC-conjugated goat anti-mouse IgG. After incubation, the cells were rewashed and stained with DAPI for 10 min. Cells were covered with a coverslip and photographed under a fluorescent microscope (BX51, Olympus, Tokyo, Japan).

### Matrigel Based Cord Formation Assay

Cells were cultured in a Matrigel basement layer. Cells were grown previously up to the desired confluency. Cells were pretreated for 24 h with the desired treating agent according to previously calculated IC_50_ value, i.e., IL-1β (15 ng/ml), SB203580 (10 μM for MCF-7 cells and 7 μM for MDA-MB-231 cells), GDC-0941 (5 nM for MCF-7 cells and 3 nM for MDA-MB-231 cells). Matrigel basement layer was first established in a 96-well plate by diluting the growth factor, reduced Matrigel with 1:1 ratio in serum-free DMEM, and 50 μl of the mixture was added in each well. After the plate was incubated at 37°C for 30 min to form a gel layer, the differently treated cells were seeded accordingly, and the results were assessed after the indicated time period. Phase-contrast images were taken under a light microscope (Olympus, Tokyo, Japan).

### Protein-Protein Interaction (PPI)

The Search Tool for the Retrieval of Interacting Genes (STRING) was used for identifying the interacting proteins whose expressions have been obtained via experiments. The possible nodes and edges of high significant proteins were further applied for Gene Ontology (GO) pathway enrichment analysis for the classification based on biological processes.

### Statistical Analysis

Experiments were carried out at least three times. The data were statistically analyzed by GraphPad Prism. Student *t*-test was performed for single grouped experiments, and One-way ANOVA or Two-way ANOVA was performed for multiple group comparisons followed by Sidak's, Tukey's, and Dunnett's multiple comparisons test according to data sets and variables. For the statistical evaluations of blot results, the intensity was measured with image-J software relative to the intensity of the concerned GAPDH to determine the actual fold expression. Statistical analysis of VM was performed by taking an average of counted intersections at three different random fields in three different independent groups using image-J software.

## Results

### IL-1β Upregulates VM Biomarkers in Breast Cancer Cells and Xenograft Tissue

**Figure 1:**
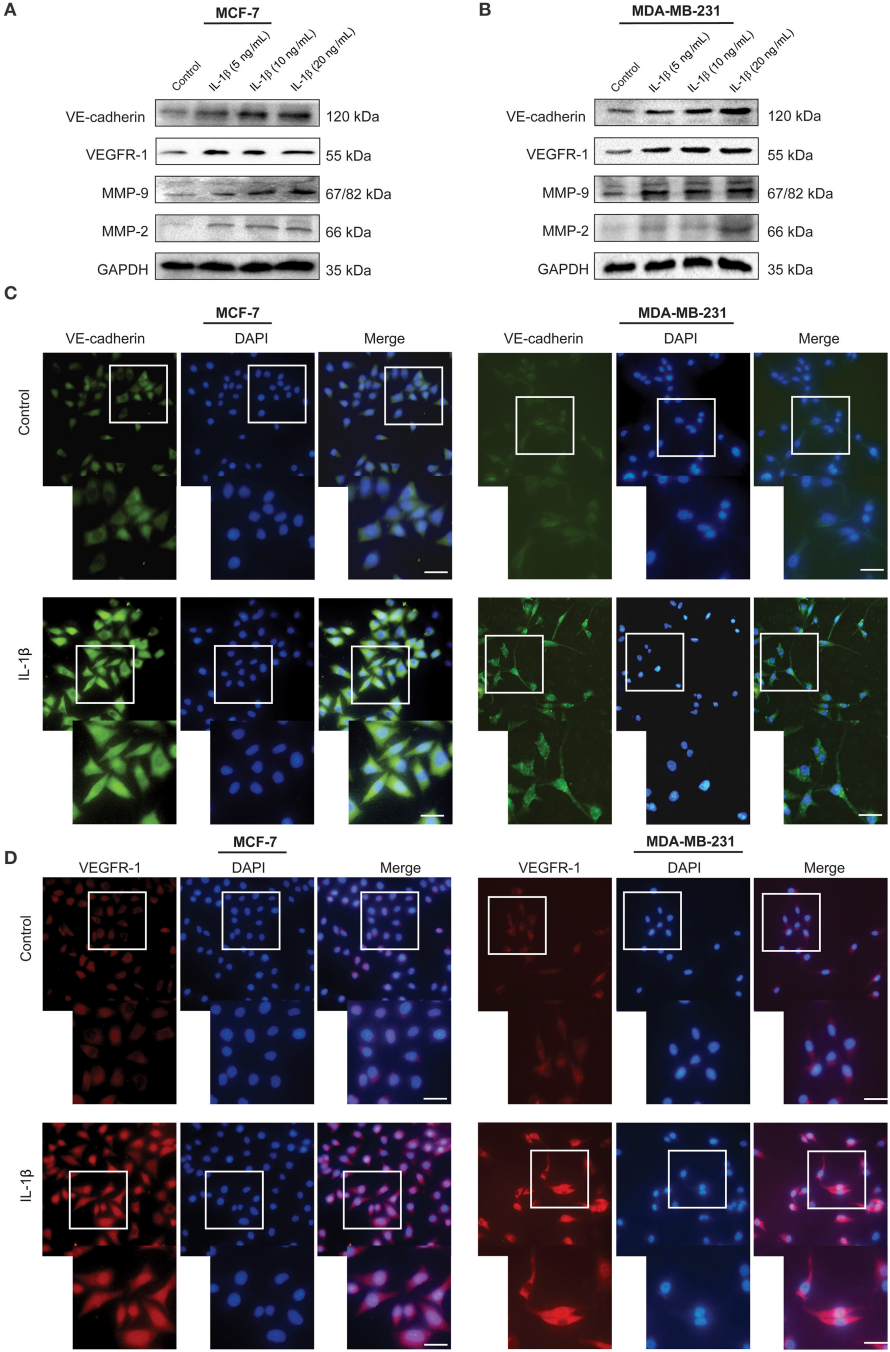
VM biomarkers are highly expressed in IL-1β treated breast cancer cells. **(A,B)** Western blot analysis of VM biomarkers (VE-cadherin, VEGFR-1, MMP-9, and MMP-2) in IL-1β (0, 5, 10, and 20 ng/ml) treated MCF-7 and MDA-MB-231 cells. **(C,D)** Immunofluorescent staining of VE-cadherin and VEGFR-1 in IL-1β (15 ng/ml) treated MCF-7 and MDA-MB-231 cells. Scale bar: 20 μm.

**Figure 2:**
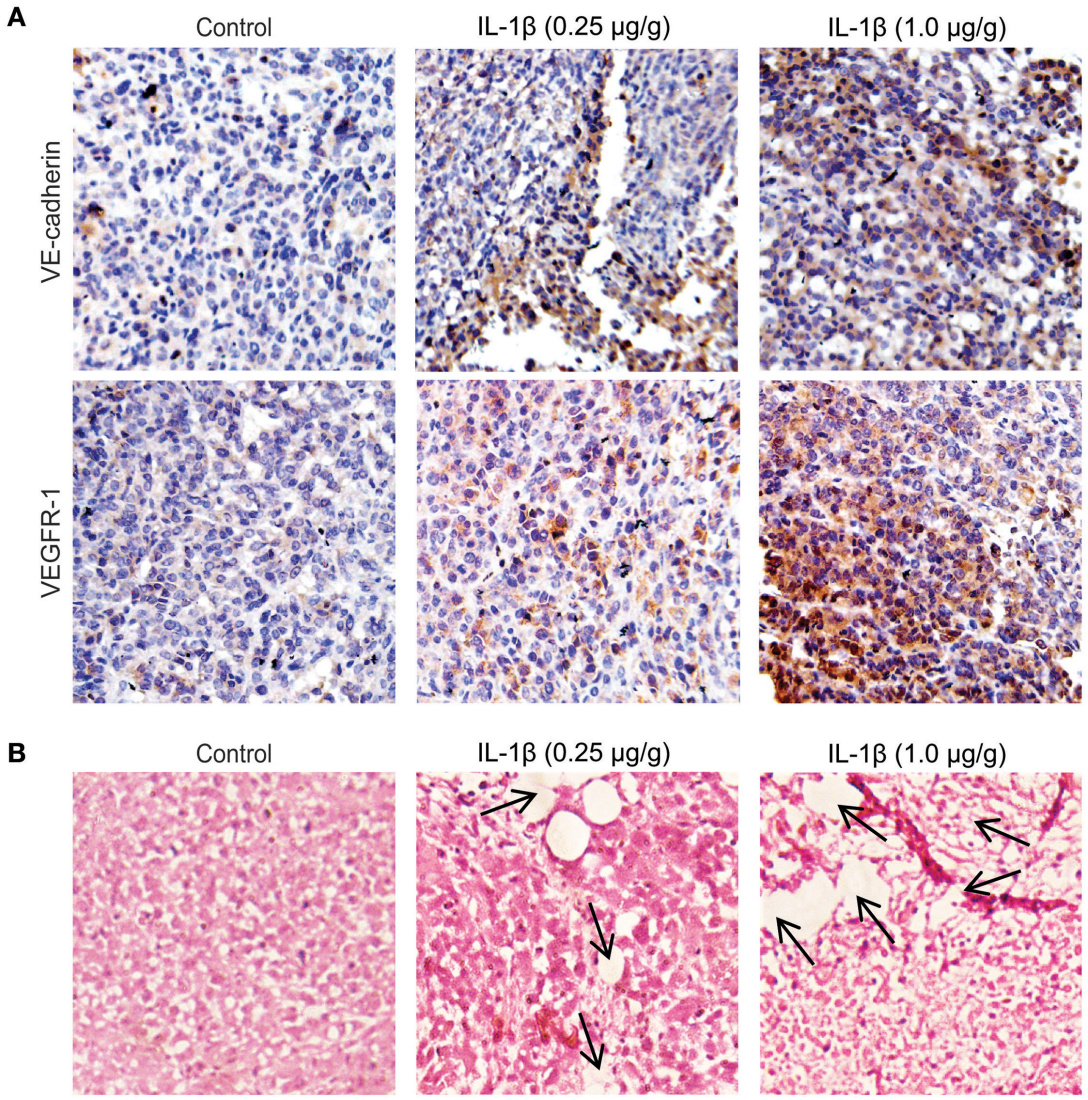
VM biomarkers and microvessel formation increases in IL-1β treated breast cancer tissues of mice models. **(A)** Immunohistochemical staining for the analysis of the expression of VE-cadherin and VEGFR-1 in the paraffine-embedded tumor slides (4 μm) IL-1β (0.25 and 1 μg/g) treated xenografts and control. **(B)** Analysis of microvessel formation (indicated by arrows) by hematoxylin and eosin staining in control and in IL-1β (0.25 and 1 μg/g) treated tumor models (Magnification 10X).

To investigate the role of IL-1β in the promotion of VM-associated events, we first detected the expression of VM biomarkers, such as VE-cadherin, VEGFR-1, MMP-9, and MMP-2. MCF-7 and MDA-MB-231 breast cancer cells were treated with IL-1β (5, 10, and 20 ng/ml) for 24 h. Western blot results showed that the expression of VE-cadherin, VEGFR-1, MMP-9, and MMP-2 was increased in both breast cancer cells (Figures 1A,B). The two key biomarkers (VE-cadherin and VEGFR-1) were further evaluated via immunofluorescent staining in breast cancer cells. Higher expression of VE-cadherin and VEGFR-1 was observed in IL-1β treated MCF-7 and MDA-MB-231 breast cancer cells (Figures 1C,D). Moreover, the IHC results showed an increase in the VM biomarkers (VE-cadherin and VEGFR-1) in the xenografts obtained from IL-1β (1 and 0.25 μg/g/day) treated mice (Figure 2A). Furthermore, the H&E staining showed an increase in microvessel formation in xenograft tissues obtained from mice treated with IL-1β as compared to the control group (Figure 2B). These results suggest a positive influence of IL-1β on the expression of VM biomarkers in breast cancer cells.

### IL-1β Enhances VM Responses in Normoxic and Hypoxic Conditions

**Figure 3:**
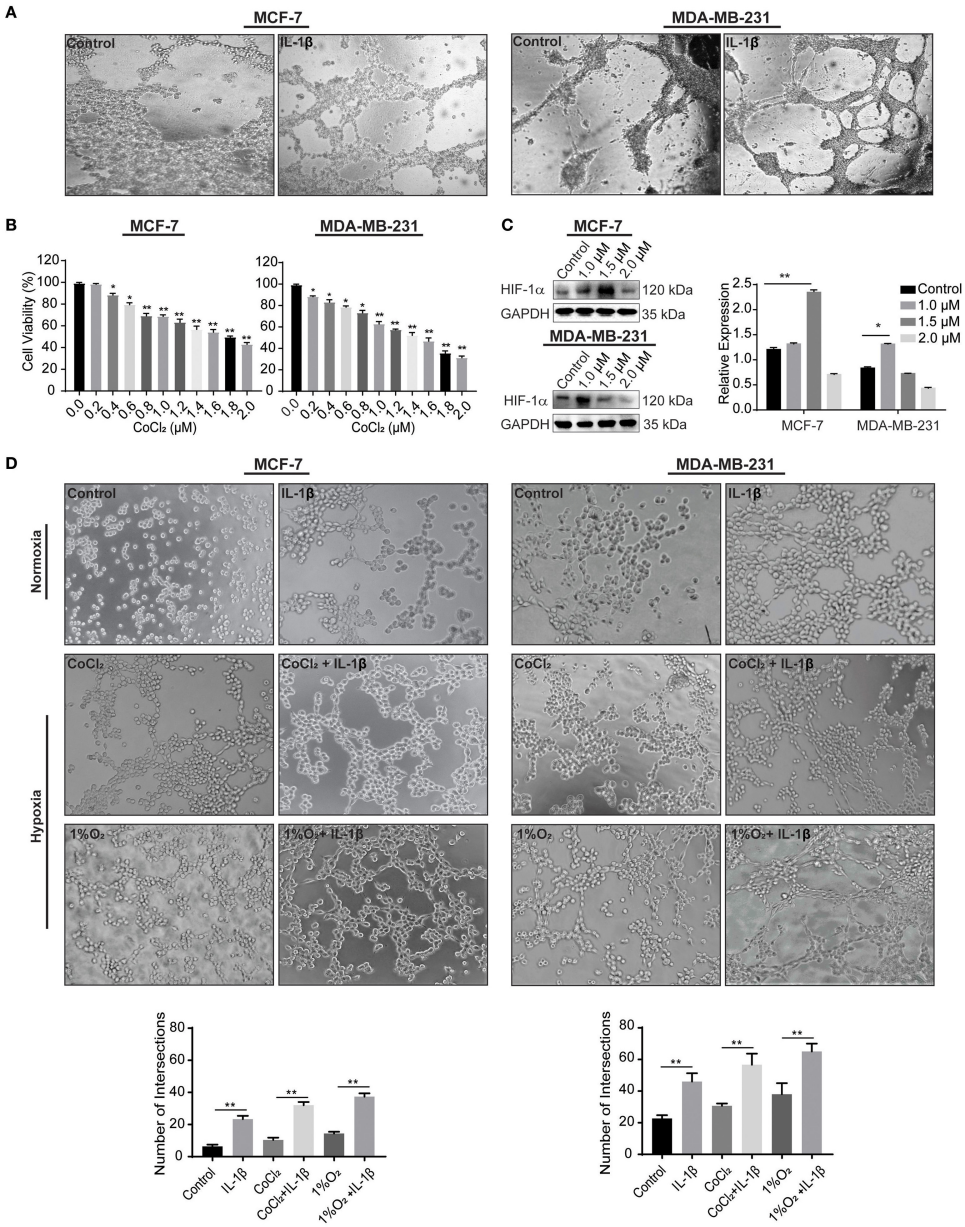
IL-1β promotes the microvessel like intersections in normoxia and hypoxia. **(A)** Matrigel based tube formations assay for the evaluations of intersections formed in response to IL-1β (15 ng/ml) in MCF-7 and MDA-MB-231 breast cancer cells at 48 h. **(B)** Cytotoxicity of CoCl_2_ was evaluated by CCK-8 assay in cells treated with different concentrations of CoCl_2_ (0, 0.2, 0.4, 0.6, 0.8, 1.0, 1.2, 1.4, 1.6, 1.8, and 2.0 μM), and One-way ANOVA was performed following Sidak's multiple comparisons with *p <0.05, **p <0.01 vs. control group. **(C)** Western blot analysis of HIF-1α expression in CoCl_2_ treated breast cancer cells. **(D)** Cord formation assay was performed to evaluate intersections in cells treated with IL-1β (15 ng/ml) in normoxic and hypoxic conditions (CoCl_2_ and 1% O_2_). Statistical analysis was performed through multiple *t*-test comparisons with *p <0.05, **p <0.01 compared to the control.

We first acquired the resulted vessel-like intersection after 48 h of treatment with IL-1β and showed a significant increase in the number of intersections compared to the control (Figure 3A). Hypoxia is a well-known factor for both classical angiogenesis and VM in the tumor microenvironment (22). Hypoxic conditions were further given to the breast cancer cells. Since the hypoxia induces autophagic reactions, the Matrigel-based assay was transformed in order to acquire the evidence of VM by analyzing the cord formation at the time of 2–4 h and seeding a reduced number of cells. The leading agent for hypoxia mediated VM is HIF-1α, which is involved in the regulation of VM related biomarkers, such as VE-cadherin and VEGFR-1. CoCl_2_ has also been studied to stimulate HIF-1α (23). In the current study, we used CoCl_2_ and 1%O_2_ to induce a hypoxic condition as a confirmatory step toward the induction of cord formation on Matrigel. Before performing *in vitro* reconstruction of Matrigel-based cord formation, we first identified the IC_50_ of CoCl_2_ via cytotoxicity assay using CCK-8 kit on MCF-7 and MDA-MB-231 cells (Figure 3B). The calculated IC_50_ for MCF-7 was 1.7 and 1.3 μM for MDA-MB-231 breast cancer cells. Using the range of concentration of CoCl_2_ (0, 1, and 2 μM), the expression of HIF-1α was also assessed in breast cancer cells by western blot (Figure 3C). The data showed that CoCl_2_ promotes HIF-1α expression in both breast cancer cells. Based on the IC_50_ and HIF-1α change, we determined that the most appropriate concentrations of CoCl_2_ were 1.5 and 1 μM for MCF-7 and MDA-MB-231 cells, respectively.

The results from 2 to 4 h of Matrigel-based cord formation assay also showed an increase in the number of intersections in breast cancer cells treated with IL-1β compared with the control stimulations. Hypoxic conditions (CoCl_2_ and 1% O_2_) enhances the effect of IL-1β in breast cancer cells (Figure 3D). MCF-7 cells had poor cord formation compared to MDA-MB-231 cells, but the number of intersections was increased with the IL-1β treatment in comparison with the control of hypoxic and normoxic conditions. MDA-MB-231, on the other hand, has an aggressive nature and provided dense intersections with the treatment of IL-1β. These results provide evidence of the stimulatory role of IL-1β toward the induction of VM-associated events in breast cancer cells.

### IL-1β Facilitates VM Responses via p38/MAPK and PI3K/Akt Signaling Pathways

**Figure 4:**
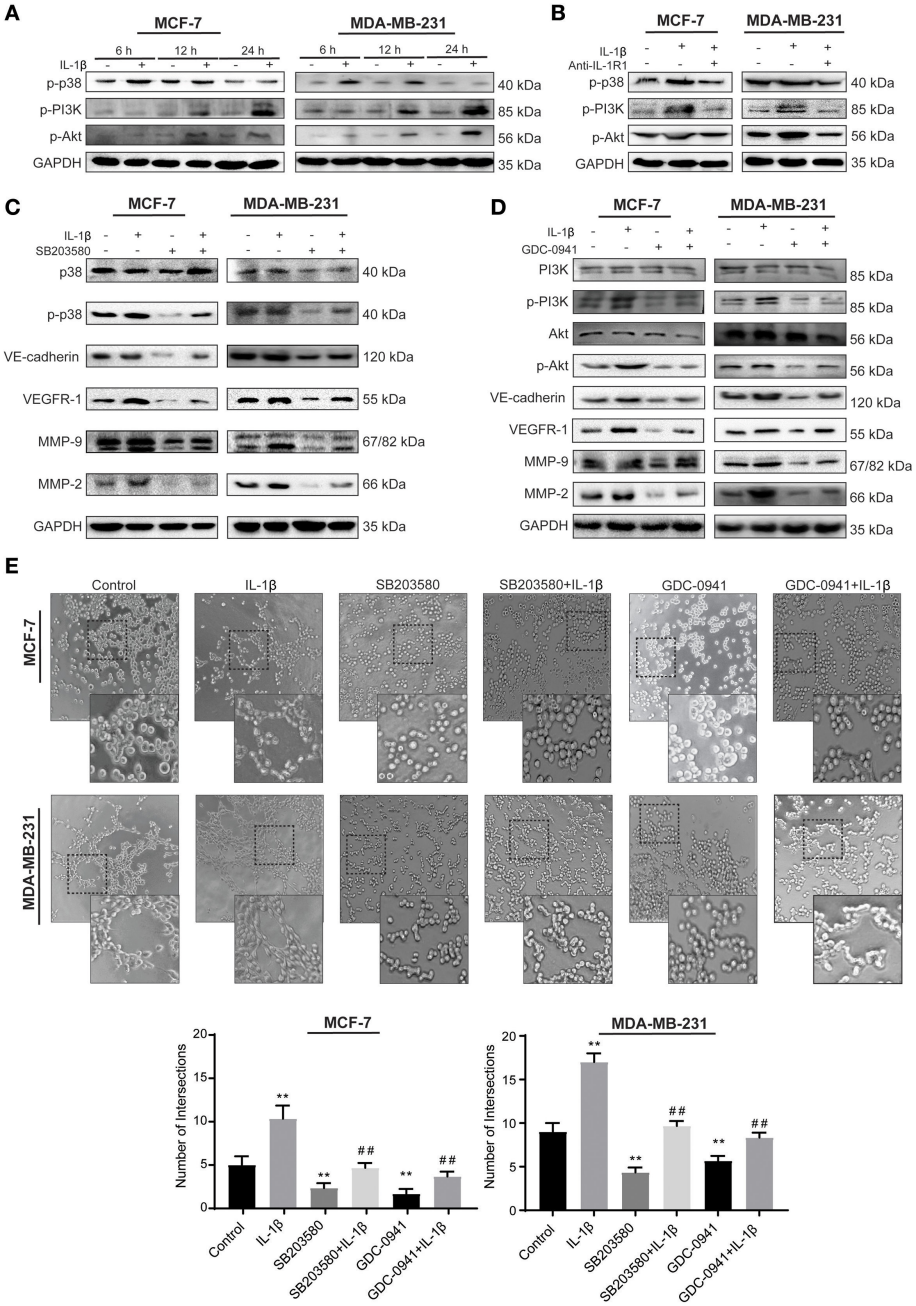
IL-1β facilitates VM responses via p38/MAPK and PI3K/Akt signaling pathways. **(A)** Time-dependent analysis of p38/MAPK and PI3K/Akt signaling pathways activation via western blot after cells treated with IL-1β. **(B)** p-p38, p-PI3K, and p-Akt treated by IL-1R1 is analyzed via western blot in cells treated with IL-1β and IL-1R1 antibody (1 μg/ml). **(C)** Expression of VM biomarkers in cells treated with p38/MAPK inhibitor (SB203580, 10 μM for MCF-7 cells, 7 μM for MDA-MB-231cells), and IL-1β. **(D)** Expression of VM biomarkers in cells treated with PI3K/Akt inhibitor (GDC-0941, 5 nM for MCF-7 cells, 3 nM for MDA-MB-231 cells) and IL-1β. **(E)** Cord formation assay was performed in cells treated with SB203580 or GDC-0941. Statistical analysis was performed through multiple *t*-test comparisons with significance of **p <0.01 compared to the control group, where as ^##^*p* <0.01 represent the statistical comparison with the IL-1β treated group. Statistical analysis of **(A–D)** is represented in Supplementary Figure 2.

To explore whether p38/MAPK and PI3K/Akt signaling pathways mediate the effect of IL-1β, we detected the activation of p-p38, p-PI3K, and p-Akt after IL-1β treatment for 6, 12, and 24 h to analyze the time-dependent effect of IL-1β on these signaling pathways. The results showed the higher activation of p-p38 at early stages (6–12 h) after treatment, while PI3K/Akt activation was detected at 12–24 h (Figure 4A). Based on the fact that IL-1β usually exerts its function by binding to IL-1 receptor 1 (IL-R1), IL-R1 antibody was used to block the binding of IL-1β to IL-R1. These results showed that IL-1β activated the p38/MAPK and PI3K/Akt signaling pathways, while IL-1R1 antibody significantly reduced the p38/MAPK and PI3K/Akt signaling as compared to the IL-1β treated group (Figure 4B). Furthermore, inhibition of p38/MAPK and PI3K/Akt signaling pathways were also assessed by using p38/MAPK inhibitor (SB203580, 10 μM for MCF-7 cells, 7 μM for MDA-MB-231 cells) and PI3K/Akt inhibitor (GDC-0941, 5 nM for MCF-7 cells, 3 nM for MDA-MB-231 cells), and the changes of VM related biomarkers (VE-cadherin, VEGFR-1, MMP-9, and MMP-2) were detected (Figures 4C,D). We found that the inhibiiton of PI3K/Akt downregulated VE-cadherin, MMP-9, and MMP-2. There was no significant change in the expression of VEGFR-1 after 24 h of treatment (Supplementary Figure 3C). However, with the 48 h of GDC-0941 treatment, the expression of VEGFR-1 was significantly reduced in breast cancer cells (Figure 4D). These western blot results showed that IL-1β enhanced the activation of the p38/MAPK and PI3K/Akt in breast cancer cells, and the addition of the inhibitors detained the stimulation. The inactivation of p38/MAPK and PI3K/Akt also inhibited the expression of VM-related markers.

The effect of IL-1β, SB203580, and GDC-0941 on VM biomarkers provided significant evidence that they are involved in the initiation of microtransformation in breast cancer cells. Therefore, we then pretreated breast cancer cells with IL-1β, SB203580, and GDC-0941 alone and in combination for Matrigel based cord formation assay. We evaluated the alterations in intersections formed in the presence of the inhibitors (Figure 4E). As shown in Figure 4E, IL-1β induced cord formations, while the inhibitors partly arrested the effect of IL-1β. These results indicate that the p38/MAPK and PI3K/Akt signaling pathways activated by IL-1β play a critical role in these morphological changes, leading to VM responses. Moreover, we can conclude that the expression of VE-cadherin, MMP-9, and MMP-2 is dependent on p38/MAPK and PI3K/Akt pathway simultaneously, while VEGFR-1 expression depends on p38/MAPK directly and have indirect relation with PI3K/Akt through IL-1β, which is also crucial in breast cancer cells progression.

### IL-1β Promotes the Activation of AP-1 Complex Involving p38/MAPK and PI3K/Akt Signaling Pathways

**Figure 5:**
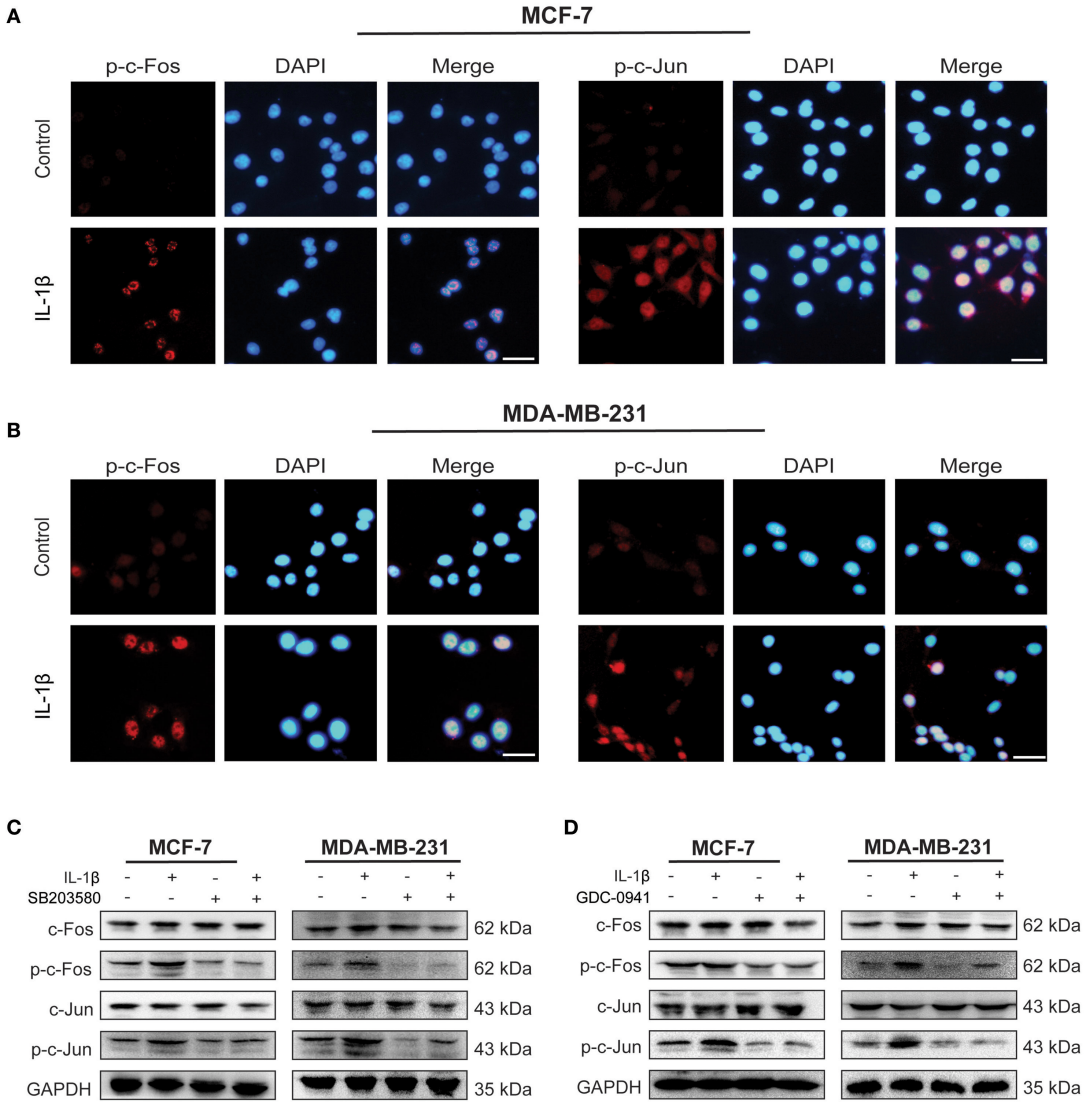
IL-1β activates AP-1 complex (c-Fos/c-Jun) in breast cancer cells. **(A,B)** Immunofluorescent staining of p-c-Fos and p-c-Jun in cells treated with IL-1β (Scale bar: 20 μm). **(C)** Western blot analysis of AP-1 complex (c-Fos/p-c-Fos and c-Jun/p-c-Jun) after cells treated with IL-1β and SB203580. **(D)** Western blot analysis of AP-1 complex in cells treated with IL-1β and GDC-0941. Statistical analysis **(C,D)** is represented in Supplementary Figure 2.

As an essential transcription factor, AP-1 complex initiates the expression of many stress-related genes (24). The effect of IL-1β on AP-1 activation was detected by immunofluorescent staining and western blot in MCF-7 and MDA-MB-231 cells (Figure 5). As shown in Figures 5A,B, more p-c-Fos and p-c-Jun were accumulated on the nuclei of both breast cancer cells after IL-1β treatment, compared with the untreated control breast cancer cells indicating upregulated p-c-Fos and p-c-Jun. Our results also confirmed that IL-1β could activate the AP-1 complex via p38/MAPK and PI3K/Akt signaling pathways. The inhibition of p38/MAPK via SB203580 after treatment for 24 h or PI3K/Akt via GDC-0941 for 48 h reduced the activation of p-c-Fos and p-c-Jun (Figures 5C,D). However, 24 h of GDC-0941 treatment did not significantly change the activation of AP-1 (Supplementary Figure 3C). These results show that the IL-1β mediated activation of the AP-1 complex is associated directly with p38/MAPK signaling pathway. These results indicates that p38/MAPK and PI3K/Akt, both of the signaling pathways, have the potential to regulate AP-1.

### Protein-Protein Interaction (PPI) and GO Enrichment Analysis of the Interacting Molecules Stimulated by IL-1β

**Figure 6:**
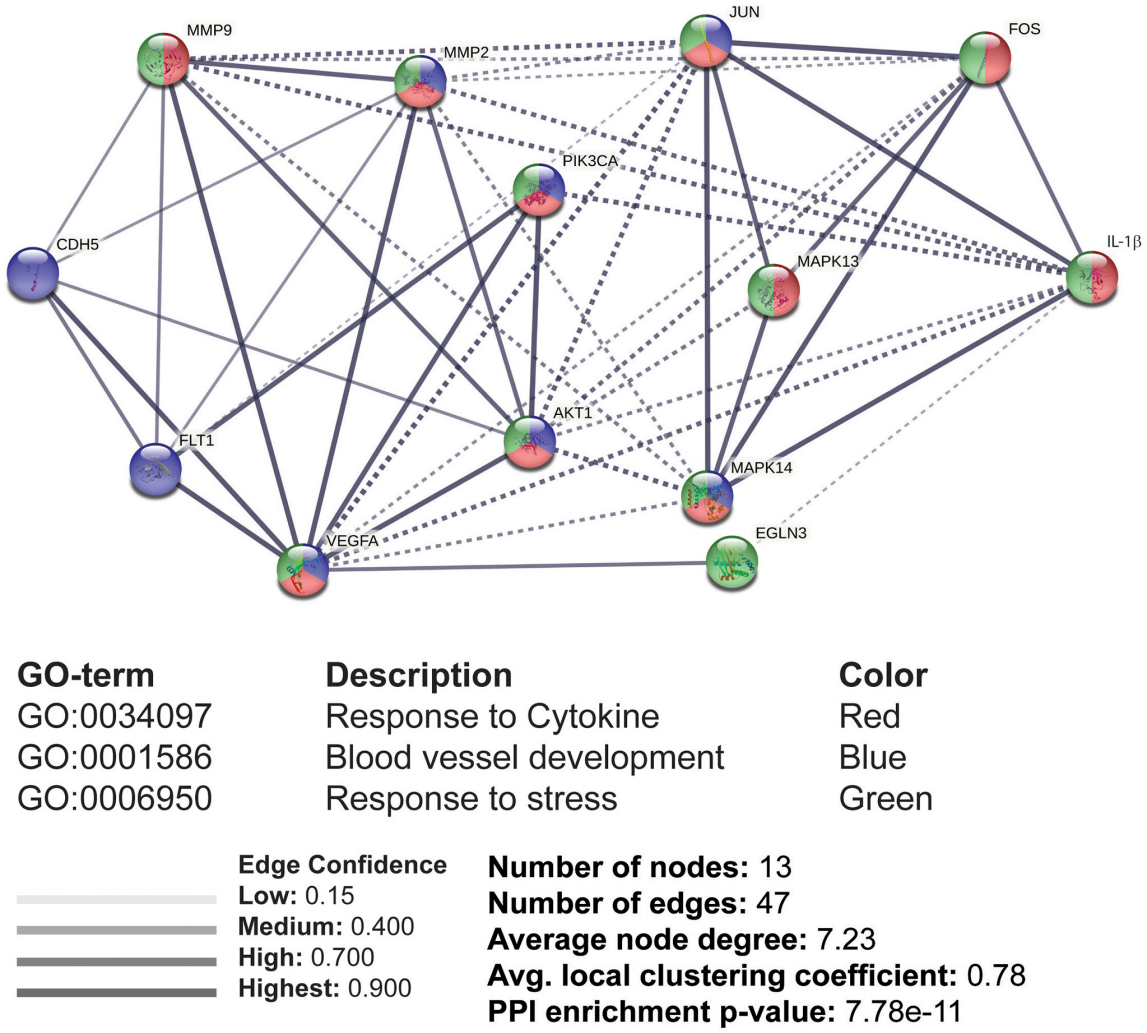
The interacting molecules stimulated by IL-1β through protein-protein interaction (PPI) network and Gene Ontology (GO) enrichment analysis. PPI and GO enrichment analysis of selected proteins were performed via the STRING online tool. Dashed lines represent the interaction between different clusters, while solid lines represent the interacting molecules within the same cluster. Colors (red, blue, and green) were provided to classify the concerning biological processes, including responses to cytokines, blood vessel development, and response to stress.

The reported data supplied the available information to generate a PPI network for designing a pathway map. We used STRING tool to evaluate the protein interactions, which were later assessed for GO enrichment analysis under the biological processes (BP). A total of 13 nodes and 47 edges were formed with significant confidence having a *p*-value of 7.78e-11. The proteins were divided into three clusters for the group classification based on function. The GO enrichment of these proteins was under the BP, including responses to cytokines, blood vessel development, and stress (Figure 6). The molecular interactions were monitored based on the confidence level of the PPI network. MAPK13 (p38), Akt, c-Jun, c-Fos, MMP-9, and MMP-2 were identified to be stimulated by IL-1β, which was supportive of our experimental results.

## Discussion

Cytokines have been reported to play a role that may favor the angiogenesis (2). In melanoma, the autocrine secretion of VEGF-A is shown to activate PI3K/PKCα and integrin signaling pathway downstream VEGFR-1, thereby leading to VM (25). TGF-β promotes epithelial-mesenchymal transition, cell migration, and VM in osteosarcoma (26). Studies established that the interleukin family can mediate angiogenesis and VM (27). IL-6 has been studied extensively in VM promotion and crosslinked to STAT3 in human hepatocellular carcinoma (28). Other IL family members such as IL-4 and IL-8 are also associated with macrophage-dependent VM promotion (29, 30). The inhibition of TGF-β signaling inhibits VM along with the inhibition of IL-4 and IL-6 in addition to IFN-γ (31). Studies propose an association between increased IL-1β expression, breast cancer metastasis, and bone microvasculature. In IL-1β deficient mice and using IL-1RA in wild type, an antagonist for IL-1β, the vascularization of the tumor was abrogated (32). Inhibiting the IL-1β pathway reduces stepwise metastasis of breast cancer models (33). These data suggest that cytokines have a strong correlation with angiogenic and VM responses. Together with our results, it is evident that the IL-1β initiates vascular events, which can advance cancer cells to undergo VM.

Tumor micro vascularization is the initial phase that leads to migration and metastasis. Its regulation depends upon a number of factors (34). Except growth factors, hypoxia, cytokines, and hormones, microvascularization behavior may also vary based on the type and origin of cancer cells. Based on cell physiology, not every type of cancer cell can transform into microvessel formation. For instance, previous studies in various cancer cell types revealed different microvascular morphologies, depending on the shape and length of intersections and cross-sections (35–38). VM has been detected in many aggressive cancer types, and several models have been used, such as ovarian cancer, melanoma cancer, and breast cancer, to study the fundamental nature of VM (3). MDA-MB-231 breast cancer cells are mostly being used as a potential model for the study of VM for triple-negative breast cancers (36, 37). The capability of VM formation is considered more common in triple-negative breast cancer. Triple-positive breast cancer type, on the other hand, is not well-known for the capability of VM formation. Under normal circumstances, MCF-7 breast cancer cells did not form cord networks efficiently. However, by stimulating MCF-7 with IL-1β and hypoxia, significant intersections were detected when cells were seeded on Matrigel. Together with the expression of VM-associated biomarkers, these findings suggest that the VM formation should not be considered exclusively for triple-negative breast cancer.

Oxygen-deprived conditions are key microenvironmental changes that promotes progression in several tumor models such as glioma, breast cancer, melanoma, oral cancer, and salivary adenoid cystic carcinoma (39–43). The VM formation in ovarian cancer cells increased metastasis potential in hypoxia (44). HIF-1α is an essential transcriptional factor response to hypoxia by regulating the corresponding gene expressions, especially VM-related molecules, including VEGF, MMPs, and LOX (31, 45). It was reported that the inhibition of HIF-1α interfered the VM formation by downregulating VE-cadherin (46, 47). Hypoxic conditions are commonly achieved by provision of 1% oxygen (48). Apart from the traditional method for the creation of hypoxic conditions, we also induced hypoxia by adding CoCl_2_, and confirmed it by expressing HIF-1α in breast cancer cells. Additionally, the intersections were increased in hypoxia as compared with normoxia, adding to the evidence that hypoxia also contributes to microvascular changes stimulated by IL-1β.

**Figure 7:**
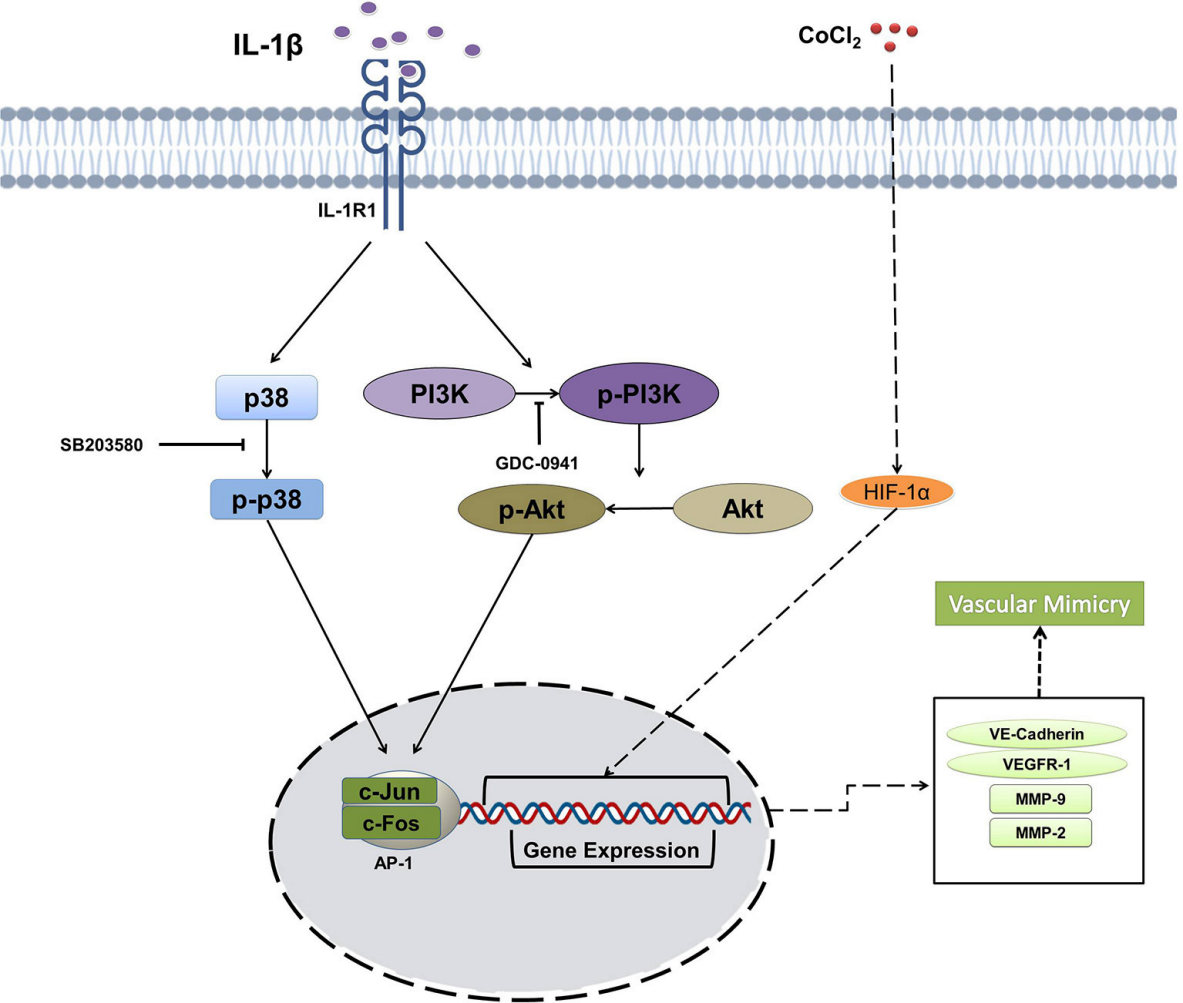
Schematic illustration summarizing the effect of IL-1β on the vasculogenic mimicry (VM) of breast cancer cells via p38/MAPK and PI3K/Akt signaling pathways.

Previous reports have revealed that many signaling pathways mediate VM in some cancer cells. PI3K/PKCα and FAK signaling pathways were activated in melanoma, thereby leading to VM (25). Wnt/β-catenin signaling was involved in VM formation in colon cancer (49). Recently, microarray analysis showed the enriched TGF-β and Wnt signaling pathways during the osteosarcoma VM (26). p38/MAPK and PI3K/Akt signaling pathways are involved in the inflammation and angiogenesis of cancer, especially in VM. For example, VM formation was increased through activating PI3K/Akt/MMPs pathway and inducing the EMT process in hepatocellular carcinoma (50). p38/MAPK signaling pathway participates in VM formation in SHG44 glioma cells *in vitro* and *in vivo* (51). Our results showed that IL-1β promoted VM formation by activating p38/MAPK and PI3K/Akt signaling pathways, and the inactivation of which by the respective inhibitors impaired the VM formation, as illustrated in Figure 7. Furthermore, the data from PPI network and GO enrichment analysis showed that these two signaling pathways ties their links closely with the interacting molecules upon stimulation. The data showed us that IL-1β to promote VM. However, activation of p38/MAPK signaling pathway started earlier (6–12 h) as compared with the PI3K/Akt signaling pathway (12–24 h) after adding IL-1β. The transcription factor necessary for the expression of VEGFR-1 mainly comprises AP1 complex is upregulated via the p38/MAPK signaling pathway (52). The decreased activation of AP-1 complex and VEGFR-1 was also found at later stage (48 h) after PI3K/Akt signaling pathways was inactivated by its inhibitor GDC-0941. The delay in signaling pathway and molecules indicates that there may be a crosstalk between p38/MAPK and PI3K/Akt signaling pathways mediated by VEGFR-1, and the profound mechanism requires further study.

In conclusion, IL-1β could promote the expression of VM-associated biomarkers and cord formation. The hypoxia enhanced the effect of IL-1β in breast cancer cells. VM-associated responses induced by IL-1β in breast cancer cells was mediated via p38/MAPK and PI3K/Akt signaling pathways. Targeting these pathways can reduce the VM-related events. The research provides an anti-angiogenic therapeutic strategy to reduce the malignancy of breast cancer cells by targeting the VM promoted by IL-1β.

## Data Availability Statement

The original contributions presented in the study are included in the article/Supplementary Material, further inquiries can be directed to the corresponding author/s.

## Ethics Statement

The animal study was performed by following the approved guidelines provided by Dalian Medical University's Specific Pathogen Free (SPF) Animal Care Centre.

## Author Contributions

MN: methodology, investigation, data analysis, and writing original draft. QZ and MS: review and editing. BA: investigation. MR and NT: visualization and data analysis. SU: methodology. SL and QY: supervision, review and project administration. All authors contributed to the article and approved the submitted version.

## Conflict of Interest

The authors declare that the research was conducted in the absence of any commercial or financial relationships that could be construed as a potential conflict of interest.

## Supplementary Material

Supplementary Figure 1 Statistical analysis of blots presented in Figure 1. One-way ANOVA was performed for statistical analysis of IL-1β (0, 5, 10, and 20 ng/ml) treated MCF-7 and MDA-MB-231 cells following Dunnett's multiple comparisons. ^*^*p* <0.05, ^**^*p* <0.01 vs. control group.

Supplementary Figure 2 Representation of statistical analysis of the Western blot in Figure 4. **(A–D)** Two-way ANOVA was performed for statistical analysis of Western blot results, presented in Figure 4, with ^*^*p* <0.05, ^**^*p* <0.01 compared with the control group, while ^#^*p* <0.05, ^##^*p* <0.01 represents the comparison with the IL-1β treated group.

Supplementary Figure 3 Representation of statistical analysis of the Western blot in Figure 5, and 24 h treatment of GDC-0941 for AP-1 complex analysis. **(A,B)** Two-way ANOVA was performed for statistical analysis of Western blot results, presented in Figure 5, with ^*^*p* <0.05, ^**^*p* <0.01 compared with the control group, while ^#^*p* <0.05, ^##^*p* <0.01 represents the comparison with the IL-1β treated group. **(C)** Western blot analysis of VEGFR-1 and AP-1 complex in MCF-7 and MDA-MB-231 cells treated with GDC-0941 and IL-1β for 24 h.
