# Supplementary figures and images for: IL-1β Promotes Vasculogenic Mimicry of Breast Cancer Cells Through p38/MAPK and PI3K/Akt Signaling Pathways
Source: Front Oncol. 2021 May 14;11:618839. doi: 10.3389/fonc.2021.618839 (PMC8160375; doi:10.3389/fonc.2021.618839)

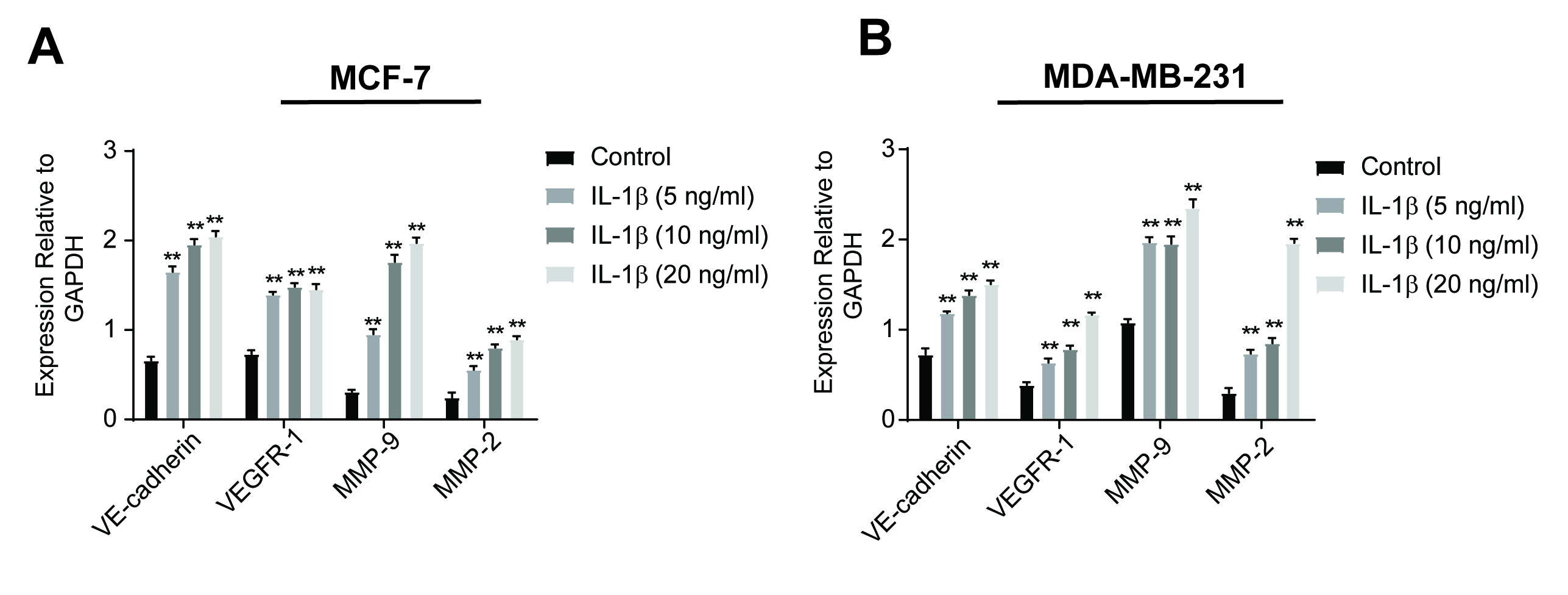

Supplement: Supplementary Figure 1 — Statistical analysis of blots presented in Figure 1. One-way ANOVA was performed for statistical analysis of IL-1β (0, 5, 10, and 20 ng/ml) treated MCF-7 and MDA-MB-231 cells following Dunnett's multiple comparisons. *p <0.05, **p <0.01 vs. control group. [file Image_1.jpg]

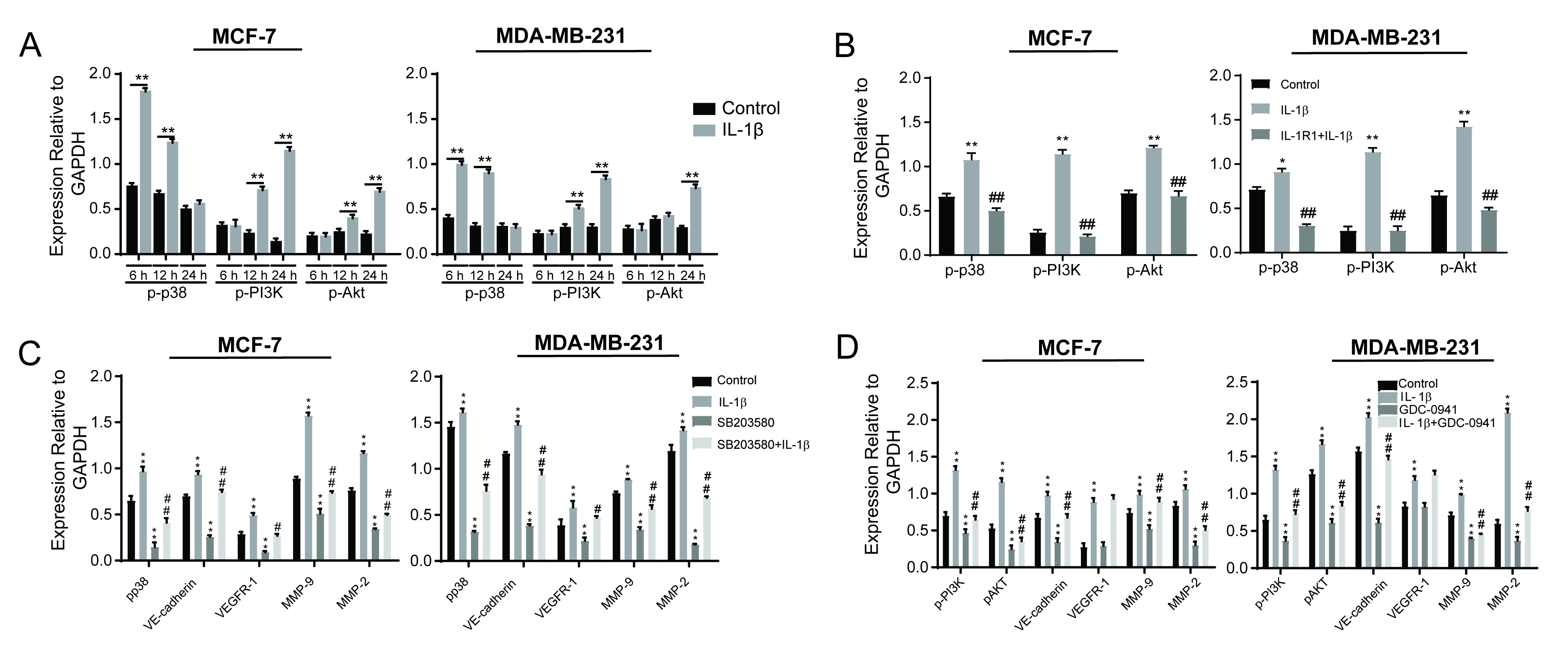

Supplement: Supplementary Figure 2 — Representation of statistical analysis of the Western blot in Figure 4. (A–D) Two-way ANOVA was performed for statistical analysis of Western blot results, presented in Figure 4, with *p <0.05, **p <0.01 compared with the control group, while #p <0.05, ##p <0.01 represents the comparison with the IL-1β treated group. [file Image_2.jpg]

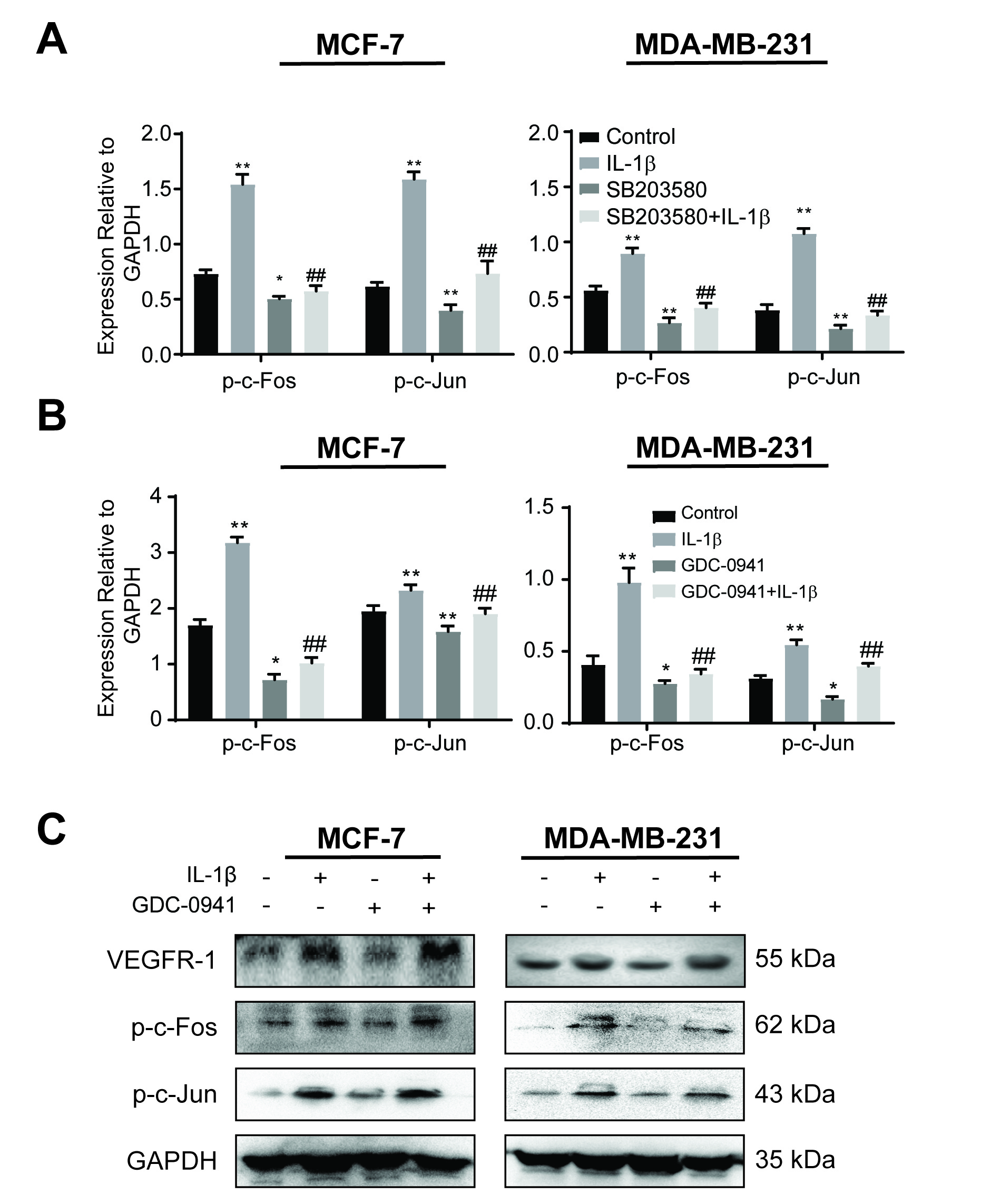

Supplement: Supplementary Figure 3 — Representation of statistical analysis of the Western blot in Figure 5, and 24 h treatment of GDC-0941 for AP-1 complex analysis. (A,B) Two-way ANOVA was performed for statistical analysis of Western blot results, presented in Figure 5, with *p <0.05, **p <0.01 compared with the control group, while #p <0.05, ##p <0.01 represents the comparison with the IL-1β treated group. (C) Western blot analysis of VEGFR-1 and AP-1 complex in MCF-7 and MDA-MB-231 cells treated with GDC-0941 and IL-1β for 24 h. [file Image_3.jpg]
